# Original research a real-world study of adverse events of nafamostat mesylate and sodium citrate based on the world health organization-VigiAccess database

**DOI:** 10.3389/fphar.2026.1795980

**Published:** 2026-05-20

**Authors:** Mengting Xu, Ziyi Wang, Binbin Yu

**Affiliations:** 1 Department of Pharmacy, The First People’s Hospital of Zhangjiagang City, Suzhou, China; 2 Department of Science and Education, The First People’s Hospital of Zhangjiagang City, Suzhou, China

**Keywords:** adverse events, extracorporeal anticoagulation, nafamostat mesylate, pharmacovigilance, sodium citrate

## Abstract

**Aims:**

While nafamostat mesylate (NM) and sodium citrate serve as commonly used extracorporeal anticoagulants in clinical practice, the characteristics and potential risks of their adverse events (AEs) need to be systematically evaluated. This study comparatively analyzed the reporting characteristics of the AEs of the above-mentioned two drugs based on the World Health Organization Adverse Drug Reaction Reporting Database (WHO-VigiAccess), to identify their safety signals and provide evidence-based guidance for optimizing their clinical uses.

**Methods:**

Mining of the global AE reporting data of NM and sodium citrate was conducted using the WHO-VigiAccess database, with the data collected until 29 December 2024. Statistical analysis was performed using the Reporting Odds Ratio (ROR), the Proportional Reporting Ratio (PRR), the Bayesian Confidence Propagation Neural Network (BCPNN), and the Empirical Bayes Geometric Mean (EBGM). The signal strengths of the AEs of the two drugs at the system organ class and preferred term levels were systematically evaluated in combination with the standardized coding MedDRA.

**Results:**

This study included 1,572 NM-related reports (59 AEs) and 485 sodium citrate-related reports (102 AEs). NM AEs were mainly concentrated in immune system diseases (23.80%), skin and subcutaneous tissue diseases (16.25%), and gastrointestinal diseases (10.10%), with strong disproportionality signals observed for thrombosis in devices (n = 18, ROR = 264.71), shock (n = 119, ROR = 186.27) and anaphylactoid shock (n = 4, ROR = 143.45). In contrast, sodium citrate-related AEs primarily included systemic diseases with various reactions at the administration site (15.75%) and gastrointestinal disorders (11.63%). The reporting proportion of mortality for sodium citrate (2.83%) was higher than that for NM, although this finding may be influenced by reporting bias and confounding by indication. In addition, this study detected signals not described in the instructions, such as citrate toxicity (ROR = 6,740.61) and spinal muscular atrophy (ROR = 665.94). Both drugs shared several high-incidence AEs, including pruritus, vomiting and dyspnea, but there are significant differences in gender, age and geographical distribution.

**Conclusion:**

The use of NM was associated with a strong disproportionality signal for severe immune-related AEs, such as anaphylactic shock, and requires strengthened monitoring. The metabolic complications of sodium citrate and its exposure risks during pregnancy require targeted optimization of medication strategies. Real-world data suggested that both drugs may cause AEs that were not mentioned in the instructions, so risk management needs to be improved through dynamic pharmacological vigilance. This study provided important references for individualized selection and safety management of anticoagulation regimens for patients requiring hemodialysis.

## 1 Introduction

The safety and efficacy of blood purification therapy are highly dependent on a rational anticoagulation strategy for preventing blood clotting during extracorporeal circulation. During extracorporeal blood purification, contact between blood and foreign surfaces in the circuit can activate the coagulation cascade, leading to coagulation within the catheter and filter. Anticoagulants are the main means of preventing such incidents. Unfractionated heparin (UFH), the most commonly used anticoagulant drug, may pose potential risks to patients, because it may induce heparin-induced thrombocytopenia (a life-threatening complication), resulting in an increased risk of bleeding (Arepally and Cines, 2020; Marini et al., 2022). Commonly used extracorporeal anticoagulants in this setting include Nafamostat mesylate (NM) and Sodium Citrate.

NM is a synthetic serine protease inhibitor that is independent of antithrombin and inhibits coagulation factors IIa, Xa, XIIa, kallikrein and hemolase, and inhibits complement and platelet activation. Currently, NM is only used as an anticoagulant in extracorporeal circulation during continuous renal replacement therapy (CRRT), extracorporeal membrane oxygenation, hemodialysis, or anticoagulation therapy in patients with left ventricular assist devices (Kamijo et al., 2020). NM is the preferred anticoagulant for critically ill, bleeding, and postoperative patients, and the use of NM has significantly reduced the incidence of bleeding complications from 64% to 4% (Lang et al., 2022). However, despite its remarkable efficacy, clinical studies and case reports suggest that it may cause hyperkalemia, bleeding tendencies, allergic reactions and even acute kidney injury, cardiopulmonary arrest, etc (Boehm et al., 2022; Shioya et al., 2022; Miao and Chen, 2025).

Sodium citrate is a local anticoagulant with a negative charge. It blocks the conversion of prothrombin to thrombin by chelating calcium ions, achieves its anticoagulant effect through multiple steps in the coagulation cascade. It is commonly used in CRRT, blood preservation, and blood transfusion (Chen et al., 2024). Compared with heparin, sodium citrate has a more selective anticoagulant effect and a lower risk of bleeding, so it is widely used in patients with renal dysfunction. However, its metabolic complications (such as hypocalcemia, metabolic alkalosis) and potential arrhythmia risks (such as QT interval prolongation) still require great attention (Xun et al., 2021; Ho et al., 2022; Di Mario et al., 2023).

Adverse event (AE) is a significant factor affecting the safety of clinical drug use, particularly in critically ill patients and those undergoing long-term treatment. The occurrence of AEs may lead to treatment discontinuation, worsening of the disease, or even death. Therefore, pharmacovigilance (PV) of drugs after marketing is crucial. Real-world data (RWD) has become an important supplement to pharmacological vigilance research due to its broad population coverage and its indication of actual medication use and effects (Lee et al., 2022). Among them, the Spontaneous Reporting System (SRS), as the core tool for AE monitoring, can identify potential drug risks early and provide a basis for clinical decision-making (Jiao et al., 2024). At present, safety studies on NM and sodium citrate are mainly based on clinical trials and single center retrospective analyses, while large-scale real-world AE data analyses remain relatively scarce. Clinical trials are often constrained by sample size, strict inclusion and exclusion criteria, and short observation periods, making it difficult to comprehensively assess the safety of drugs in complex clinical settings. In contrast, SRSs can integrate AE data globally, providing early warnings for rare or long-term medication risks.

The WHO-VigiAccess database is maintained by the WHO Uppsala Monitoring Centre, which includes AE reports from over 200 countries. It is currently one of the largest drug safety monitoring platforms in the world. This database uses Medical Dictionary for Drug Regulatory Activities (MedDRA) terminology to standardize the coding of AEs, facilitating cross-regional and cross-population data mining. In recent years, studies based on the VigiAccess database have found that the most common ocular AEs of four anti-VEGF drugs primarily involve visual impairment, blurred vision, and blindness, with persistent differences in AEs among different drugs (Li et al., 2024). Together, these findings demonstrate an important role for the VigiAccess database in pharmacological vigilance.

To provide physicians with more comprehensive safety data, and to optimize the medication strategies of NM and sodium citrate. This study aims to fill this gap by conducting the first systematic, comparative analysis of the adverse event profiles and signal strengths of these two anticoagulants using the global WHO-VigiAccess database and data mining methodologies. Our objective is not only to corroborate known risks but also to identify potential, unlabeled safety signals, thereby providing more comprehensive and precise evidence-based guidance for the individualized selection and risk management of anticoagulation regimens.

## 2 Data and methods

### 2.1 Search strategy and data source

This study systematically collected AEs of NM and sodium citrate use worldwide from the WHO-VigiAccess database as of 29 December 2024, by accessing https://www.vigiaccess.org. No filters were applied regarding age, sex, region, or report year, thus a total sampling technique was adopted. Data retrieval was performed using a Python script to query the platform’s backend. The collected data included the patient’s region, sex, age, reporting year, as well as the number of reports on drug-related AEs categorized by system organ class (SOC) and the preferred term (PT) of specific symptoms. All drugs in the study were identified by their generic names, and their terms were mapped using the MedDRA version 27.0. The WHO-VigiAccess database is a spontaneous reporting system that primarily collects information on the occurrence of AEs associated with drug use. However, it does not systematically record key temporal variables such as the duration of drug exposure, cumulative dose, or time-to-event. Therefore, the present retrospective pharmacovigilance analysis identifies AE signals based on the number of reported cases and disproportionality metrics, without being able to assess associations with treatment duration or dose-response relationships.

### 2.2 Disproportionality analysis

Disproportionation analysis is a data mining method that is mainly used to evaluate the correlation between drugs and adverse reactions. The core principle involves constructing a 2 × 2 contingency table for each combination (Supplementary Table S1). A statistically significant elevation in these metrics suggests a potential safety signal, warranting further clinical investigation. To evaluate AE signals associated with NM and sodium citrate, we employed four disproportionality analysis methods: the Reporting Odds Ratio (ROR), the Proportional Reporting Ratio (PRR), the Bayesian Confidence Propagation Neural Network (BCPNN), and the Empirical Bayes Geometric Mean (EBGM). The specific threshold criteria for each method were as follows:

ROR required number of reports (a) ≥ 3, ROR ≥1 and the lower limit of the 95% confidence interval (CI) > 1;
PRR required a ≥3, PRR ≥2, and the lower limit of the 95% CI > 1 or χ2≥4 ;
BCPNN required the lower limit of the 95% credibility interval for the Information Component (IC_025_) > 0;
EBGM required the lower limit of the 95% confidence interval for the Empirical Bayes Geometric Mean (EBGM_05_) > 2. Specific methods are described in Supplementary Table S2.
A higher value for the lower limit of the 95% CI indicates a stronger signal strength, meaning a more robust association between the target drug and the relevant AE (Zou et al., 2024; Fan et al., 2025).

It should be noted that disproportionality analyses (ROR, PRR, BCPNN, and EBGM) are univariate methods that do not adjust for potential confounders, such as age, sex, concomitant medications, underlying diseases, or indication severity. Therefore, the detected signals represent crude statistical associations and should be interpreted as hypothesis-generating rather than causal.

### 2.3 Statistical analyses

This study was designed using a retrospective descriptive analysis. Descriptive statistical analysis was performed using Microsoft Excel to characterize the patients who experienced AEs associated with NM and sodium citrate. The AE reporting rate for each symptom was defined as its reported frequency divided by the total AE reports for the respective drug. We used ratios and percentages to classify descriptive variables.

## 3 Results

### 3.1 Basic information of cases of reported AEs related to NM and sodium citrate

This study collected a total of 2,057 reports, including 1,572 on NM and 485 on sodium citrate. In terms of sex, NM-related AEs were reported in a higher proportion of males than females. In contrast, sodium citrate-related AEs were reported in a slightly higher proportion of females than males, while the difference between the two sexes was relatively small. In terms of age, NM-related AEs were reported mainly in individuals aged 45–74 years, corresponding to middle-aged and elderly patients. In comparison, sodium citrate-related AEs were reported primarily among individuals aged 18–44 years, with 21.44% of these reports lacking age information. In terms of regional distribution, reports on NM-related AEs were mainly from Asia, while sodium citrate-related AEs were predominantly reported from the Americas and Europe. The earliest adverse reaction reports in the WHO-VigiAccess database were recorded in 1988 for NM and 1981 for sodium citrate. Over the past 12 years, the number of reported AEs related to NM was higher in 2024 and 2013 than in other years, while the number of reported AEs related to sodium citrate was higher in 2019 and 2022 than in other years. The data are detailed in Table 1.

**TABLE 1:** Characteristics of AEs reports of nafamostat mesylate and sodium citrate.

Characteristics	Nafamostat mesylate [n (%)]	Sodium citrate [n (%)]
Sex		
Female	633 (40.27)	229 (47.22)
Male	902 (57.38)	206 (42.47)
Unknown	37 (2.35)	50 (10.31)
Age		
0–27 days	5 (0.32)	2 (0.41)
28 days to 23 months	2 (0.13)	5 (1.03)
2–11 years	5 (0.32)	15 (3.09)
12–17 years	9 (0.57)	4 (0.82)
18–44 years	142 (9.03)	170 (35.05)
45–64 years	465 (29.58)	112 (23.09)
65–74 years	465 (29.58)	39 (8.04)
75 years	395 (25.13)	34 (7.01)
Unknown	84 (5.34)	104 (21.44)
Continent		
Africa	0 (0.00)	4 (0.82)
Americas	2 (0.13)	215 (44.33)
Asia	1,567 (99.68)	55 (11.34)
Europe	2 (0.13)	196 (40.41)
Oceania	1 (0.06)	15 (3.09)
Report year		
1981	0 (0.00)	1 (0.21)
1983	0 (0.00)	1 (0.21)
1988	2 (0.13)	3 (0.62)
1989	0 (0.00)	22 (4.54)
1990	0 (0.00)	1 (0.21)
1991	3 (0.19)	0 (0.00)
1992	5 (0.32)	1 (0.21)
1993	1 (0.06)	1 (0.21)
1994	1 (0.06)	1 (0.21)
1996	2 (0.13)	1 (0.21)
1998	4 (0.25)	0 (0.00)
1999	0 (0.00)	1 (0.21)
2000	0 (0.00)	1 (0.21)
2001	4 (0.25)	2 (0.41)
2002	0 (0.00)	2 (0.41)
2004	0 (0.00)	2 (0.41)
2005	2 (0.13)	11 (2.27)
2006	27 (1.72)	13 (2.68)
2007	12 (0.76)	0 (0.00)
2008	0 (0.00)	5 (1.03)
2009	54 (3.44)	4 (0.82)
​2010	61 (3.88)	5 (1.03)
2011	18 (1.15)	12 (2.47)
2012	27 (1.72)	10 (2.06)
2013	157 (9.99)	17 (3.51)
2014	127 (8.08)	9 (1.86)
2015	111 (7.06)	21 (4.33)
2016	131 (8.33)	23 (4.74)
2017	138 (8.78)	16 (3.3)
2018	121 (7.7)	35 (7.22)
2019	98 (6.23)	80 (16.49)
2020	37 (2.35)	41 (8.45)
2021	30 (1.91)	43 (8.87)
2022	5 (0.32)	44 (9.07)
2023	60 (3.82)	24 (4.95)
2024	334 (21.25)	32 (6.6)

### 3.2 AE distribution at the system-organ class (SOC) level

**FIGURE 1:**
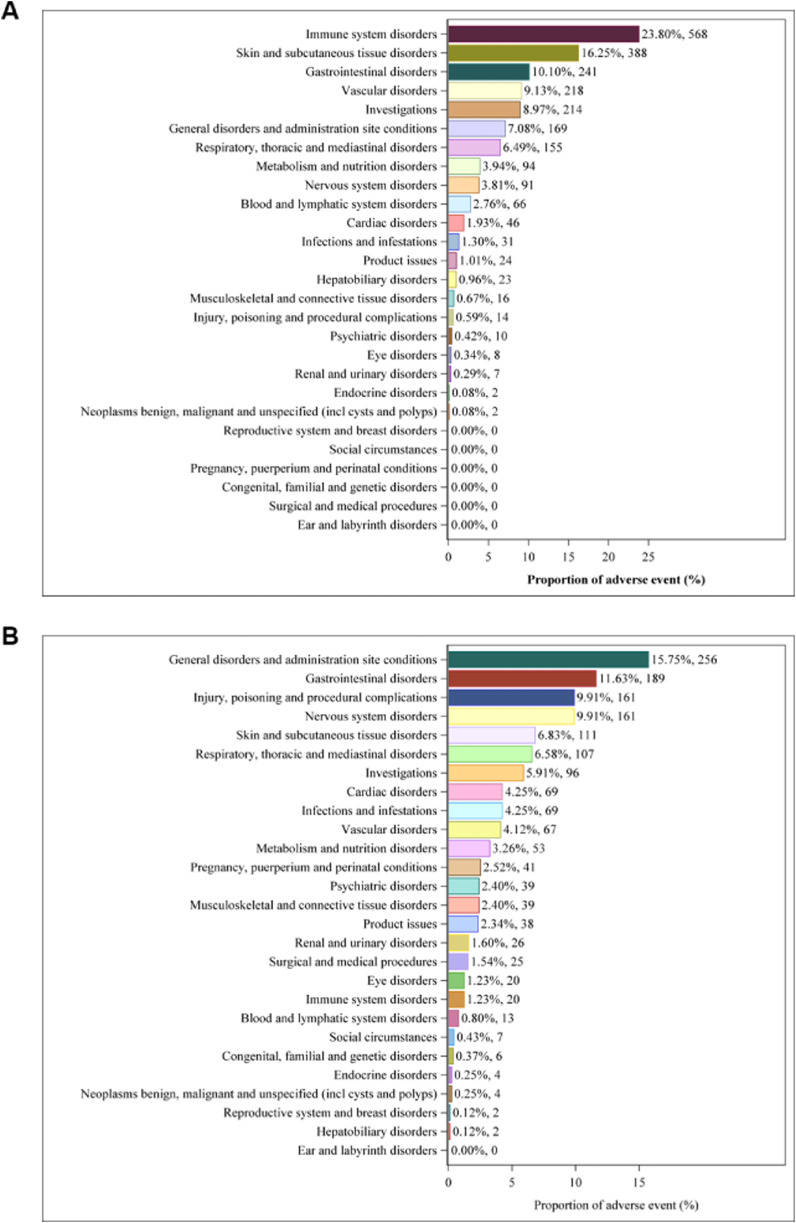
Proportion of adverse events by SOCs. **(A)** Nafamostat mesylate; **(B)** sodium citrate.

Figures 1A,B show the percentages of AE signals associated with NM and sodium citrate at the SOC level, respectively. A total of 21 SOCs related to NM and 26 SOCs related to sodium citrate were identified. Specifically, the reported incidences of NM-related AEs, such as immune system disorders (23.80%, n = 568), skin and subcutaneous tissue disorders (16.25%, n = 388), and vascular disorders (9.13%, n = 218), were higher than those of sodium citrate-related AEs. In contrast, the reported incidences of sodium citrate-related AEs, including systemic diseases and various reactions at the administration site (15.75%, n = 256), gastrointestinal disorders (11.63%, n = 189), injury poisoning and procedural complications (9.91%, n = 161), were higher than those of NM-related AEs.

### 3.3 AE distribution at the PT level

**FIGURE 2:**
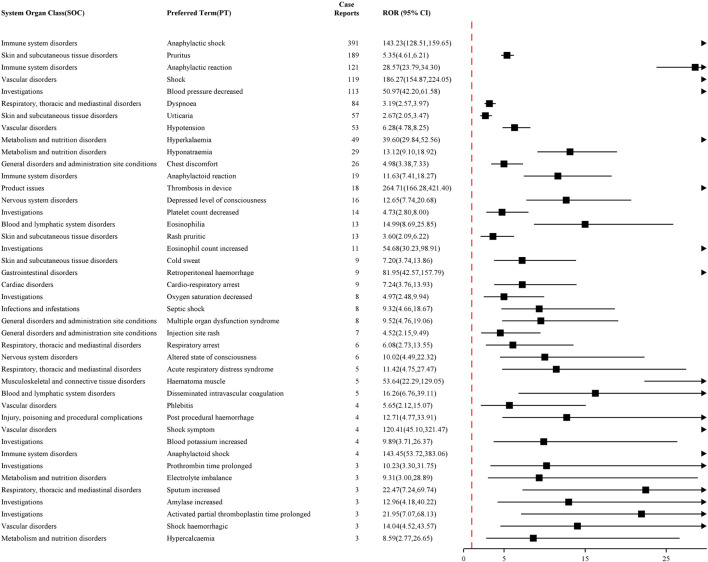
Top 50 PTs in terms of signal frequency of nafamostat mesylate.

**FIGURE 3:**
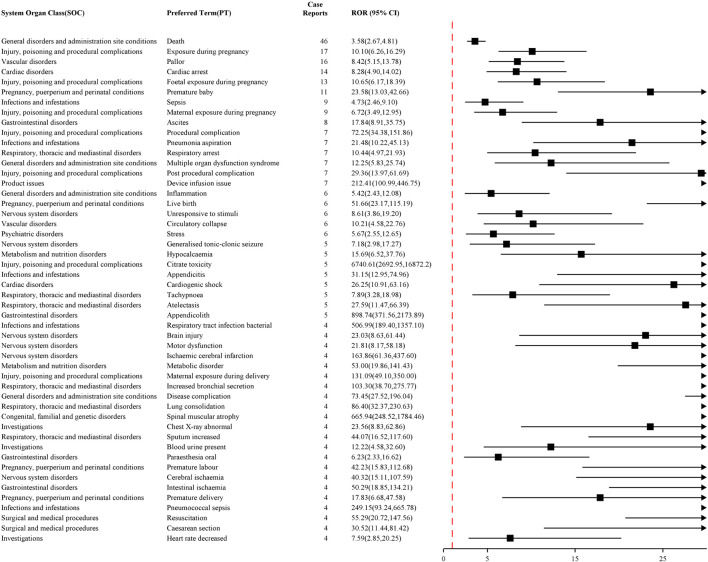
Top 50 PTs in terms of signal frequency of sodium citrate.

The top 50 PTs with AEs frequencies for NM and sodium citrate are reported in Figures 2, 3, respectively. Commonly reported AE symptoms shared by the two drugs included pruritus, vomiting, nausea, dyspnea, and rash. While a relatively high proportion of deaths was reported for sodium citrate (2.83%, n = 46), no fatal cases were reported for NM. Anaphylactic shock was reported much more frequently for NM (16.38%, n = 391) than for sodium citrate. Additionally, disproportionate reporting was observed for NM with symptoms such as thrombosis in device (0.75%, n = 18), thrombocytopenia (0.59%, n = 14), eosinophilia (0.54%, n = 13), and tachycardia (0.38%, n = 9). Fetal (0.80%, n = 13) and maternal (0.55%, n = 9) exposure during pregnancy, as well as premature delivery (0.68%, n = 11), were reported for sodium citrate. In contrast, no such symptom was reported for NM. Supplementary Tables S3, S4, respectively, provide detailed explanations for AE signals of NM and sodium citrate that meet four algorithmic criteria simultaneously at the PT level. Among them, shock, allergic symptoms, increased eosinophil count, decreased platelet count, and hyperkalemia were consistent with those described in the instructions for NM, while hypocalcemia, metabolic disorders, and muscle atrophy aligned with the instructions for sodium citrate.

**FIGURE 4:**
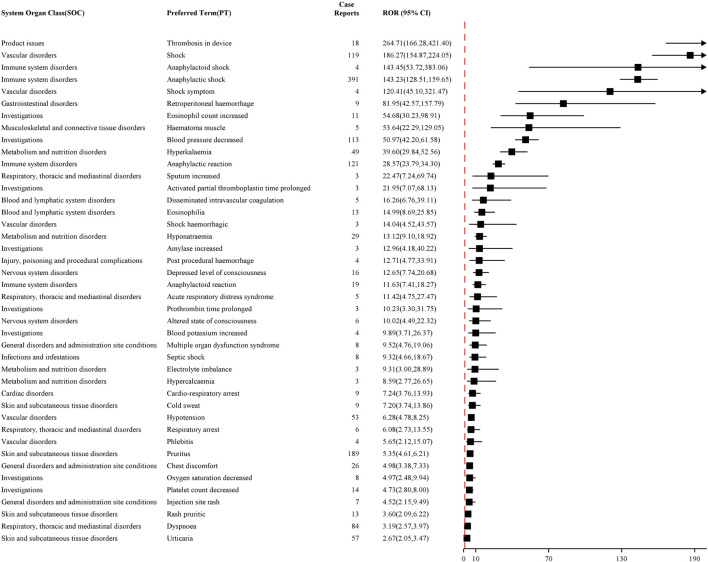
Top 50 PTs in terms of signal intensity of nafamostat mesylate.

**FIGURE 5:**
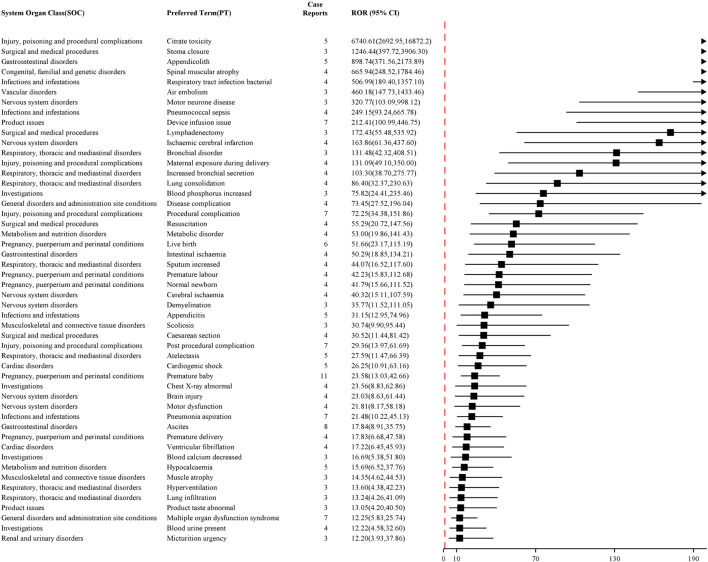
Top 50 PTs in terms of signal intensity of sodium citrate.

Furthermore, the top 50 PTs were ranked based on the intensity of signals that were simultaneously positive across all four Algorithms. As shown in Figures 4, 5, the top five PTs with the highest signal intensities for NM were thrombosis in device (ROR = 264.71, n = 18), shock (ROR = 186.27, n = 119), anaphylactoid shock (ROR = 143.45, n = 4), anaphylactic shock (ROR = 143.23, n = 391), and shock symptom (ROR = 120.41, n = 4). The top five PTs with the highest signal intensities for sodium citrate were citrate toxicity (ROR = 6,740.61, n = 5), stoma closure (ROR = 1,246.44, n = 3), appendicolith (ROR = 898.74, n = 5), spinal muscular atrophy (ROR = 665.94, n = 4), and bacterial respiratory tract infection (ROR = 506.99, n = 4). Tables 2, 3 present the high-strength AE signals for NM and sodium citrate, respectively, which were concurrently identified by all four algorithms at the Preferred Term level. The signals are ranked by ROR values. For AEs with case numbers ≤5 (e.g., anaphylactoid shock, sputum increased, hypercalcaemia), the corresponding signal estimates should be interpreted with caution due to potential instability and susceptibility to reporting bias.

**TABLE 2:** Signal strength of adverse events at the PT level for nafamostat mesylate ranked by ROR.

System organ class (SOC)	Preferred term (PT)	Case reports	ROR (95% CI)	PRR (95% CI)	Chi Square	IC (IC025)	EBGM (EBGM05)
Product issues	Thrombosis in device	18	264.71 (166.28,421.40)	262.72 (165.61,416.78)	4,668.38	8.03 (3.49)	261.34 (164.16)
Vascular disorders	Shock	119	186.27 (154.87,224.05)	177.04 (148.54,210.99)	20,761.4	7.46 (5.89)	176.41 (146.67)
Immune system disorders	Anaphylactoid shock	4	143.45 (53.72,383.06)	143.21 (53.71,381.80)	563.23	7.16 (0.99)	142.80 (53.47)
Immune system disorders	Anaphylactic shock	391	143.23 (128.51,159.65)	119.94 (109.53,131.33)	46,067.4	6.90 (6.36)	119.65 (107.34)
Vascular disorders	Shock symptom	4	120.41 (45.10,321.47)	120.21 (45.10,320.41)	471.73	6.91 (0.98)	119.92 (44.92)
Gastrointestinal disorders	Retroperitoneal haemorrhage	9	81.95 (42.57,157.79)	81.65 (42.51,156.81)	715.80	6.35 (2.26)	81.52 (42.34)
Investigations	Eosinophil count increased	11	54.68 (30.23.98.91)	54.43 (30.18.98.19)	576.39	5.76 (2.48)	54.38 (30.06)
Musculoskeletal and connective tissue disorders	Haematoma muscle	5	53.64 (22.29,129.05)	53.53 (22.29,128.55)	257.46	5.74 (1.28)	53.47 (22.22)
Investigations	Blood pressure decreased	113	50.97 (42.20.61.58)	48.61 (40.60.58.20)	5,269.08	5.60 (4.82)	48.56 (40.20)
Metabolism and nutrition disorders	Hyperkalaemia	49	39.60 (29.84.52.56)	38.81 (29.41.51.21)	1804.54	5.28 (4.05)	38.78 (29.22)
Immune system disorders	Anaphylactic reaction	121	28.57 (23.79.34.30)	27.17 (22.84.32.33)	3,054.35	4.76 (4.21)	27.16 (22.62)
Respiratory, thoracic and mediastinal disorders	Sputum increased	3	22.47 (7.24.69.74)	22.44 (7.24.69.56)	61.44	4.49 (0.37)	22.43 (7.23)
Investigations	Activated partial thromboplastin time prolonged	3	21.95 (7.07.68.13)	21.93 (7.07.67.95)	59.89	4.45 (0.37)	21.92 (7.06)
Blood and lymphatic system disorders	Disseminated intravascular coagulation	5	16.26 (6.76.39.11)	16.23 (6.76.38.96)	71.44	4.02 (1.02)	16.22 (6.75)
Blood and lymphatic system disorders	Eosinophilia	13	14.99 (8.69.25.85)	14.91 (8.67.25.64)	168.73	3.90 (2.13)	14.91 (8.64)
Vascular disorders	Shock haemorrhagic	3	14.04 (4.52.43.57)	14.02 (4.53.43.46)	36.28	3.81 (0.28)	14.02 (4.52)
Metabolism and nutrition disorders	Hyponatraemia	29	13.12 (9.10.18.92)	12.97 (9.03.18.63)	320.62	3.70 (2.68)	12.97 (8.99)
Investigations	Amylase increased	3	12.96 (4.18.40.22)	12.95 (4.18.40.12)	33.06	3.69 (0.25)	12.94 (4.17)
Injury, poisoning and procedural complications	Post procedural haemorrhage	4	12.71 (4.77.33.91)	12.70 (4.77.33.80)	43.09	3.67 (0.63)	12.69 (4.76)
Nervous system disorders	Depressed level of consciousness	16	12.65 (7.74.20.68)	12.57 (7.71.20.49)	170.46	3.65 (2.20)	12.57 (7.69)
Immune system disorders	Anaphylactoid reaction	19	11.63 (7.41.18.27)	11.55 (7.38.18.07)	183.16	3.53 (2.27)	11.55 (7.35)
Respiratory, thoracic and mediastinal disorders	Acute respiratory distress syndrome	5	11.42 (4.75.27.47)	11.40 (4.75.27.36)	47.43	3.51 (0.88)	11.40 (4.74)
Investigations	Prothrombin time prolonged	3	10.23 (3.30.31.75)	10.22 (3.30.31.67)	24.95	3.35 (0.18)	10.22 (3.29)
Nervous system disorders	Altered state of consciousness	6	10.02 (4.49.22.32)	9.99 (4.49.22.22)	48.56	3.32 (1.04)	9.99 (4.48)
Investigations	Blood potassium increased	4	9.89 (3.71.26.37)	9.87 (3.71.26.28)	31.89	3.30 (0.54)	9.87 (3.70)
General disorders and administration site conditions	Multiple organ dysfunction syndrome	8	9.52 (4.76.19.06)	9.49 (4.75.18.96)	60.80	3.25 (1.32)	9.49 (4.74)
Infections and infestations	Septic shock	8	9.32 (4.66.18.67)	9.30 (4.65.18.57)	59.24	3.22 (1.31)	9.29 (4.64)
Metabolism and nutrition disorders	Electrolyte imbalance	3	9.31 (3.00.28.89)	9.30 (3.00.28.82)	22.22	3.22 (0.15)	9.30 (3.00)
Metabolism and nutrition disorders	Hypercalcaemia	3	8.59 (2.77.26.65)	8.58 (2.77.26.58)	20.08	3.10 (0.12)	8.58 (2.76)
Cardiac disorders	Cardio-respiratory arrest	9	7.24 (3.76.13.93)	7.21 (3.76.13.85)	48.19	2.85 (1.24)	7.21 (3.75)
Skin and subcutaneous tissue disorders	Cold sweat	9	7.20 (3.74.13.86)	7.18 (3.74.13.78)	47.89	2.84 (1.23)	7.18 (3.73)
Vascular disorders	Hypotension	53	6.28 (4.78.8.25)	6.16 (4.72.8.04)	230.03	2.62 (2.09)	6.16 (4.69)
Respiratory, thoracic and mediastinal disorders	Respiratory arrest	6	6.08 (2.73.13.55)	6.07 (2.73.13.49)	25.40	2.60 (0.72)	6.07 (2.72)
Vascular disorders	Phlebitis	4	5.65 (2.12.15.07)	5.64 (2.12.15.03)	15.29	2.50 (0.26)	5.64 (2.12)
Skin and subcutaneous tissue disorders	Pruritus	189	5.35 (4.61.6.21)	5.01 (4.37.5.74)	615.77	2.32 (2.08)	5.01 (4.32)
General disorders and administration site conditions	Chest discomfort	26	4.98 (3.38.7.33)	4.94 (3.37.7.24)	81.80	2.30 (1.55)	4.94 (3.35)
Investigations	Oxygen saturation decreased	8	4.97 (2.48.9.94)	4.95 (2.48.9.89)	25.25	2.31 (0.82)	4.95 (2.47)
Investigations	Platelet count decreased	14	4.73 (2.80.8.00)	4.71 (2.79.7.94)	40.93	2.23 (1.17)	4.71 (2.78)
General disorders and administration site conditions	Injection site rash	7	4.52 (2.15.9.49)	4.51 (2.15.9.45)	19.14	2.17 (0.63)	4.51 (2.15)
Skin and subcutaneous tissue disorders	Rash pruritic	13	3.60 (2.09.6.22)	3.59 (2.09.6.17)	24.32	1.84 (0.82)	3.59 (2.08)
Respiratory, thoracic and mediastinal disorders	Dyspnoea	84	3.19 (2.57.3.97)	3.11 (2.52.3.84)	121.97	1.64 (1.28)	3.11 (2.51)
Skin and subcutaneous tissue disorders	Urticaria	57	2.67 (2.05.3.47)	2.63 (2.04.3.40)	58.19	1.40 (0.97)	2.63 (2.02)

Ranked by ROR.

Signals are detected when all the following criteria are met:a ≥3, PRR ≥2 and Chi-Square ≥4, lower limit of 95% CI, of ROR >1, IC025 > 0, EBGM05 > 2.

**TABLE 3:** Signal strength of adverse events at the PT level for Sodium citrate ranked by ROR.

System organ class (SOC)	Preferred term (PT)	Case reports	ROR (95% CI)	PRR (95% CI)	Chi Square	IC (IC025)	EBGM (EBGM05)
Injury, poisoning and procedural complications	Citrate toxicity	5	6,740.61 (2,692.95,16,872.1)	6,719.87 (2,691.61,16,776.8)	30,742.8	12.59 (1.35)	6,150.47 (2,457.18)
Surgical and medical procedures	Stoma closure	3	1,246.44 (397.72,3906.30)	1,244.14 (397.81,3891.01)	3,663.61	10.26 (0.54)	1,223.18 (390.30)
Gastrointestinal disorders	Appendicolith	5	898.74 (371.56,2173.89)	895.98 (371.42,2161.40)	4,415.42	9.79 (1.39)	885.07 (365.91)
Congenital, familial and genetic disorders	Spinal muscular atrophy	4	665.94 (248.52,1784.46)	664.30 (248.50,1775.80)	2,625.18	9.36 (1.01)	658.28 (245.66)
Infections and infestations	Respiratory tract infection bacterial	4	506.99 (189.40,1357.10)	505.75 (189.39,1350.51)	2001.06	8.97 (1.01)	502.25 (187.63)
Vascular disorders	Air embolism	3	460.18 (147.73,1433.46)	459.33 (147.77,1427.84)	1,363.38	8.83 (0.54)	456.45 (146.53)
Nervous system disorders	Motor neurone disease	3	320.77 (103.09,998.12)	320.18 (103.11,994.21)	950.37	8.32 (0.54)	318.78 (102.45)
Infections and infestations	Pneumococcal sepsis	4	249.15 (93.24,665.78)	248.54 (93.24,662.54)	982.83	7.95 (1.00)	247.70 (92.70)
Product issues	Device infusion issue	7	212.41 (100.99,446.75)	211.50 (100.88,443.42)	1,462.30	7.72 (1.93)	210.89 (100.27)
Surgical and medical procedures	Lymphadenectomy	3	172.43 (55.48,535.92)	172.11 (55.49,533.82)	509.16	7.42 (0.53)	171.71 (55.25)
Nervous system disorders	Ischaemic cerebral infarction	4	163.86 (61.36,437.60)	163.46 (61.35,435.48)	644.41	7.35 (0.99)	163.09 (61.07)
Respiratory, thoracic and mediastinal disorders	Bronchial disorder	3	131.48 (42.32,408.51)	131.24 (42.33,406.91)	387.04	7.03 (0.52)	131.00 (42.16)
Injury, poisoning and procedural complications	Maternal exposure during delivery	4	131.09 (49.10,350.00)	130.77 (49.09,348.31)	514.17	7.03 (0.98)	130.53 (48.89)
Respiratory, thoracic and mediastinal disorders	Increased bronchial secretion	4	103.30 (38.70,275.77)	103.05 (38.70,274.44)	403.68	6.69 (0.97)	102.91 (38.55)
Respiratory, thoracic and mediastinal disorders	Lung consolidation	4	86.40 (32.37,230.63)	86.19 (32.37,229.51)	336.43	6.43 (0.96)	86.09 (32.25)
Investigations	Blood phosphorus increased	3	75.82 (24.41,235.46)	75.68 (24.42,234.54)	220.85	6.24 (0.50)	75.60 (24.34)
General disorders and administration site conditions	Disease complication	4	73.45 (27.52,196.04)	73.27 (27.52,195.09)	284.86	6.19 (0.95)	73.20 (27.43)
Injury, poisoning and procedural complications	Procedural complication	7	72.25 (34.38,151.86)	71.95 (34.34,150.73)	489.27	6.17 (1.84)	71.88 (34.20)
Surgical and medical procedures	Resuscitation	4	55.29 (20.72,147.56)	55.16 (20.72,146.84)	212.55	5.78 (0.93)	55.12 (20.65)
Metabolism and nutrition disorders	Metabolic disorder	4	53.00 (19.86,141.43)	52.87 (19.86,140.74)	203.41	5.72 (0.92)	52.83 (19.80)
Pregnancy, puerperium and perinatal conditions	Live birth	6	51.66 (23.17,115.19)	51.47 (23.15,114.43)	296.76	5.68 (1.55)	51.44 (23.07)
Gastrointestinal disorders	Intestinal ischaemia	4	50.29 (18.85,134.21)	50.17 (18.85,133.57)	192.65	5.65 (0.92)	50.14 (18.79)
Respiratory, thoracic and mediastinal disorders	Sputum increased	4	44.07 (16.52,117.60)	43.96 (16.52,117.04)	167.86	5.46 (0.90)	43.94 (16.47)
Pregnancy, puerperium and perinatal conditions	Premature labour	4	42.23 (15.83,112.68)	42.13 (15.83,112.14)	160.52	5.40 (0.90)	42.10 (15.78)
Pregnancy, puerperium and perinatal conditions	Normal newborn	4	41.79 (15.66,111.52)	41.69 (15.66,110.98)	158.78	5.38 (0.90)	41.67 (15.62)
Nervous system disorders	Cerebral ischaemia	4	40.32 (15.11,107.59)	40.22 (15.11,107.08)	152.92	5.33 (0.89)	40.20 (15.07)
Nervous system disorders	Demyelination	3	35.77 (11.52,111.05)	35.70 (11.52,110.62)	101.15	5.16 (0.44)	35.69 (11.49)
Infections and infestations	Appendicitis	5	31.15 (12.95.74.96)	31.06 (12.94.74.54)	145.41	4.96 (1.19)	31.05 (12.90)
Musculoskeletal and connective tissue disorders	Scoliosis	3	30.74 (9.90.95.44)	30.69 (9.90.95.07)	86.13	4.94 (0.42)	30.67 (9.88)
Surgical and medical procedures	Caesarean section	4	30.52 (11.44.81.42)	30.44 (11.44.81.03)	113.86	4.93 (0.85)	30.43 (11.40)
Injury, poisoning and procedural complications	Post procedural complication	7	29.36 (13.97.61.69)	29.24 (13.96.61.24)	190.85	4.87 (1.67)	29.23 (13.91)
Respiratory, thoracic and mediastinal disorders	Atelectasis	5	27.59 (11.47.66.39)	27.51 (11.46.66.02)	127.70	4.78 (1.16)	27.50 (11.43)
Cardiac disorders	Cardiogenic shock	5	26.25 (10.91.63.16)	26.17 (10.91.62.80)	121.02	4.71 (1.15)	26.16 (10.87)
Pregnancy, puerperium and perinatal conditions	Premature baby	11	23.58 (13.03.42.66)	23.42 (13.00.42.21)	236.11	4.55 (2.19)	23.42 (12.94)
Investigations	Chest X-ray abnormal	4	23.56 (8.83.62.86)	23.51 (8.83.62.56)	86.18	4.55 (0.80)	23.50 (8.81)
Nervous system disorders	Brain injury	4	23.03 (8.63.61.44)	22.97 (8.63.61.14)	84.04	4.52 (0.80)	22.97 (8.61)
Nervous system disorders	Motor dysfunction	4	21.81 (8.17.58.18)	21.75 (8.17.57.90)	79.19	4.44 (0.79)	21.75 (8.15)
Infections and infestations	Pneumonia aspiration	7	21.48 (10.22.45.13)	21.39 (10.21.44.80)	136.04	4.42 (1.57)	21.38 (10.18)
Gastrointestinal disorders	Ascites	8	17.84 (8.91.35.75)	17.76 (8.90.35.46)	126.54	4.15 (1.67)	17.76 (8.86)
Pregnancy, puerperium and perinatal conditions	Premature delivery	4	17.83 (6.68.47.58)	17.79 (6.69.47.36)	63.39	4.15 (0.74)	17.79 (6.67)
Cardiac disorders	Ventricular fibrillation	4	17.22 (6.45.45.93)	17.18 (6.45.45.71)	60.93	4.10 (0.73)	17.17 (6.44)
Investigations	Blood calcium decreased	3	16.69 (5.38.51.80)	16.66 (5.38.51.60)	44.15	4.06 (0.32)	16.65 (5.37)
Metabolism and nutrition disorders	Hypocalcaemia	5	15.69 (6.52.37.76)	15.65 (6.52.37.55)	68.56	3.97 (1.00)	15.64 (6.50)
Musculoskeletal and connective tissue disorders	Muscle atrophy	3	14.35 (4.62.44.53)	14.32 (4.62.44.36)	37.17	3.84 (0.28)	14.32 (4.61)
Respiratory, thoracic and mediastinal disorders	Hyperventilation	3	13.60 (4.38.42.23)	13.58 (4.38.42.07)	34.96	3.76 (0.27)	13.58 (4.37)
Respiratory, thoracic and mediastinal disorders	Lung infiltration	3	13.24 (4.26.41.09)	13.22 (4.27.40.94)	33.87	3.72 (0.26)	13.21 (4.26)
Product issues	Product taste abnormal	3	13.05 (4.20.40.50)	13.02 (4.20.40.34)	33.30	3.70 (0.26)	13.02 (4.19)
General disorders and administration site conditions	Multiple organ dysfunction syndrome	7	12.25 (5.83.25.74)	12.20 (5.83.25.56)	72.00	3.61 (1.32)	12.20 (5.81)
Investigations	Blood urine present	4	12.22 (4.58.32.60)	12.19 (4.58.32.44)	41.09	3.61 (0.62)	12.19 (4.57)
Renal and urinary disorders	Micturition urgency	3	12.20 (3.93.37.86)	12.18 (3.93.37.72)	30.77	3.61 (0.24)	12.17 (3.92)
Injury, poisoning and procedural complications	Foetal exposure during pregnancy	13	10.65 (6.17.18.39)	10.58 (6.15.18.18)	112.79	3.40 (1.88)	10.58 (6.13)
Respiratory, thoracic and mediastinal disorders	Respiratory arrest	7	10.44 (4.97.21.93)	10.40 (4.96.21.78)	59.47	3.38 (1.23)	10.40 (4.95)
Vascular disorders	Circulatory collapse	6	10.21 (4.58.22.76)	10.18 (4.58.22.62)	49.66	3.35 (1.05)	10.18 (4.56)
Injury, poisoning and procedural complications	Exposure during pregnancy	17	10.10 (6.26.16.29)	10.01 (6.24.16.06)	137.93	3.32 (2.05)	10.00 (6.20)
Nervous system disorders	Unresponsive to stimuli	6	8.61 (3.86.19.20)	8.58 (3.86.19.08)	40.21	3.10 (0.95)	8.58 (3.85)
Vascular disorders	Pallor	16	8.42 (5.15.13.78)	8.35 (5.13.13.59)	103.60	3.06 (1.84)	8.35 (5.10)
Cardiac disorders	Cardiac arrest	14	8.28 (4.90.14.02)	8.22 (4.88.13.85)	88.89	3.04 (1.72)	8.22 (4.86)
Gastrointestinal disorders	Frequent bowel movements	3	7.99 (2.58.24.81)	7.98 (2.58.24.72)	18.32	3.00 (0.09)	7.98 (2.57)
Metabolism and nutrition disorders	Metabolic acidosis	3	7.98 (2.57.24.77)	7.97 (2.57.24.68)	18.28	2.99 (0.09)	7.97 (2.57)
Respiratory, thoracic and mediastinal disorders	Tachypnoea	5	7.89 (3.28.18.98)	7.87 (3.28.18.87)	29.97	2.98 (0.69)	7.86 (3.27)
Investigations	Heart rate decreased	4	7.59 (2.85.20.25)	7.57 (2.85.20.16)	22.83	2.92 (0.42)	7.57 (2.84)
Nervous system disorders	Generalised tonic-clonic seizure	5	7.18 (2.98.17.27)	7.16 (2.98.17.17)	26.50	2.84 (0.64)	7.16 (2.97)
Injury, poisoning and procedural complications	Maternal exposure during pregnancy	9	6.72 (3.49.12.95)	6.69 (3.49.12.84)	43.61	2.74 (1.18)	6.69 (3.48)
Gastrointestinal disorders	Paraesthesia oral	4	6.23 (2.33.16.62)	6.22 (2.34.16.54)	17.51	2.64 (0.31)	6.22 (2.33)
Psychiatric disorders	Stress	6	5.67 (2.55.12.65)	5.66 (2.54.12.57)	23.01	2.50 (0.67)	5.66 (2.54)
General disorders and administration site conditions	Inflammation	6	5.42 (2.43.12.08)	5.40 (2.43.12.01)	21.53	2.43 (0.64)	5.40 (2.42)
Infections and infestations	Sepsis	9	4.73 (2.46.9.10)	4.71 (2.45.9.03)	26.31	2.23 (0.86)	4.71 (2.44)
General disorders and administration site conditions	Death	46	3.58 (2.67.4.81)	3.51 (2.64.4.67)	83.28	1.81 (1.31)	3.51 (2.62)

Ranked by ROR.

Signals are detected when all the following criteria are met:a ≥3, PRR ≥2 and Chi-Square ≥4, lower limit of 95% CI, of ROR >1, IC025 > 0, EBGM05 > 2.

### 3.4 Information not included in the drug instructions

In addition to the common AEs clearly mentioned in the instructions, this study also identified suspected AEs not described in the instructions for the two drugs. For example, the reported PTs for NM included decreased blood pressure (n = 113), dyspnea (n = 84), and thrombosis in device (n = 18). The reported PTs for sodium citrate involved death (n = 46), rash (n = 26), dyspnea (n = 22), diarrhea (n = 19), and pyrexia (n = 17).

## 4 Discussion

Heparin anticoagulation therapy is the first choice for patients with thromboembolism or its related risks. NM has been used as an anticoagulant in blood purification therapy for over 30 years, demonstrating excellent *in vitro* anticoagulant properties and significant advantages for patients at risk of high bleeding. Sodium citrate anticoagulation therapy is a viable option for patients without bleeding risk or coagulopathy (Yue et al., 2024). A retrospective study compared the efficacy of NM versus sodium citrate in CRRT in children’s hospitals in Japan and the United States, showing that both agents were safe and effective for pediatric CRRT, with no difference in anticoagulant effect and safety between them (Miyaji et al., 2022). The present study systematically analyzes the real-world AE characteristics of NM and sodium citrate using the WHO-VigiAccess database, and identifies differences in safety signals and potential risks between the two drugs.

NM, a broad-spectrum synthetic serine protease inhibitor, has been widely used in Japan and South Korea for the treatment of acute pancreatitis and disseminated intravascular coagulation. It has also been approved for use in anticoagulant therapy for patients with bleeding tendencies who are undergoing CRRT or extracorporeal circulation (Hernandez-Mitre et al., 2022). Hyperkalemia is a known side effect of NM administration (Okajima et al., 2020). The mechanism underlying NM-associated hyperkalemia is thought to involve disruptions in both renal and extrarenal potassium balance. Previous studies have reported that hyperkalemia is caused by metabolites of NM rather than by the drug itself (Muto et al., 1994). The most prominent disproportionality signal for NM was observed for immune system disorders (23.80%), especially anaphylactic shock (ROR = 143.23) and anaphylactoid shock (ROR = 143.45). This statistical association aligns with previous case reports, although it does not confirm a direct causal relationship. Specifically, the NM-induced anaphylactoid reaction occurred in a hemodialysis patient after nine doses of NM (Higuchi et al., 2000), while the NM-related allergic reaction followed by persistent severe intestinal edema occurred in another patient during hemodialysis (Shindo et al., 2019). The strong signal for thrombosis in device associated with NM (ROR = 264.71) presents a paradoxical finding for an anticoagulant. A plausible mechanistic hypothesis, based on its known pharmacological profile, is that NM, as a broad-spectrum serine protease inhibitor, suppresses platelet activation. However, under the high shear stress conditions of extracorporeal circulation, excessive or aberrant inhibition of platelet function might disrupt the formation of initial platelet aggregates and prevent the establishment of a stable anticoagulant pseudointima, potentially favoring fibrin-rich thrombus formation. This hypothesis requires validation through further *in vitro* hemodynamic studies. In clinical practice, the anticoagulant effects must be weighed against the risks of immune activation. Therefore, it is recommended to enhance the monitoring of vital signs during intravenous infusion and always be prepared with emergency anti-allergy treatment plans.

Sodium citrate achieves local anticoagulation effects by chelating calcium ions, resulting in a lower bleeding risk than heparin (Zhou et al., 2023). Compared with heparin, sodium citrate has a longer filter life and superior safety performance. In recent years, it has been increasingly recommended as a first-line treatment in Europe and the United States, even among patients with a lower bleeding risk (Zarbock et al., 2020; Gonzalez-Fernandez et al., 2023; Trakarnvanich et al., 2023). However, the present study suggested that its metabolic complications (e.g., hypocalcemia and metabolic alkalosis) and pregnancy-related AEs (ROR = 9.60) need to be considered. Specifically, sodium citrate-induced hypocalcemia may trigger QT interval prolongation and arrhythmia, especially in patients with renal insufficiency (Li et al., 2022). In addition, the reported mortality for sodium citrate (2.83%) was significantly higher than that for NM; this discrepancy may be attributed to severe metabolic disorders or unidentified citrate toxicity (ROR = 6,740.61). Furthermore, this study detected extremely strong statistical signals associated with sodium citrate, such as “citrate toxicity” and “spinal muscular atrophy”. However, it is crucial to interpret these signals with caution. They represent a statistical disproportion in reporting frequency and do not necessarily confirm a direct causal relationship. These associations could be substantially confounded by factors such as: (i) confounding by indication—the underlying critical illnesses in patients requiring sodium citrate anticoagulation might themselves lead to these outcomes; (ii) concomitant medications—other drugs administered to these patients could be an explanatory factor; or (iii) reporting bias—focused or stimulated reporting of specific events might artificially inflate the signal strength. In the absence of detailed clinical records (e.g., patient history, concomitant medications), interpreting these signals as definitively “newly identified risks” is not sufficiently supported by evidence. Future research should first seek to validate the reproducibility of this signal by pooling data from multiple large pharmacoepidemiological databases. If the signal persists, subsequent basic research in cellular and animal models is warranted to investigate the potential effects of citrate or its metabolites on motor neuron survival and function.

The occurrence of shared high-incidence AEs of the two drugs, such as itching and dyspnea, may be related to histamine release or complement activation. Among these AEs, there were significant differences in sex and age distribution between the two drugs. NM-related AEs predominantly occurred in patients aged 45–74 years. Reduced immune function and increased comorbidities in the elderly population may be responsible for this phenomenon. In comparison, a higher incidence of sodium citrate-related AEs was reported in patients aged 18–44 years, involving its widespread use in females of childbearing age, including those with CRRT during pregnancy. Differences in regional distributions between the two drugs (NM reports were mainly from Asia, while those of sodium citrate were from Europe and the United States) suggest differences in medication habits and monitoring systems. This regional variation is consistent with the literature, indicating a greater utilization of NM in Asia, likely due to its perceived advantage in reducing bleeding risk (Miyaji et al., 2022).

This study identified several AEs not described in the instruction manual, such as NM-related blood pressure reduction (n = 113) and sodium citrate-related death (n = 46). While these data need to be further validated, they emphasize the value of using spontaneous reporting systems in identifying rare or long-term risks. In recent years, the role of RWD in pharmacological vigilance has become increasingly important. A systematic review emphasized that RWD can complement the shortcomings of clinical trials, especially in identifying safety signals in heterogeneous populations (Verkerk and Voest, 2024). It is therefore recommended that regulatory agencies constantly update drug instructions and promote cross-regional data sharing to optimize risk management strategies.

Based on the disproportionality signals identified in this hypothesis-generating study, we offer the following observations for future research, rather than definitive clinical recommendations. For patients with a high risk of bleeding, our data suggest that NM may be associated with a favorable safety profile due to its short half-life and low systemic bleeding risk; however, the strong immune-related signals (e.g., anaphylactic shock) warrant heightened awareness. For metabolically stable patients without severe liver impairment, sodium citrate showed signals of lower bleeding risk and potentially longer filter survival, but its metabolic complications (e.g., hypocalcemia) and pregnancy-related exposure signals require attention. Importantly, these findings are derived from spontaneous reporting data and do not establish causality. They should not replace guideline-based clinical judgment. Independent validation using other study designs (e.g., prospective cohorts or randomized trials) is necessary before any practice change can be considered.

This study has several limitations inherent to the SRS data. First, reporting biases are inevitable, including significant under-reporting (where many AEs are not identified or submitted), over-reporting (driven by increased vigilance for specific safety concerns), and notoriety bias (where public or professional attention on a drug temporarily inflates its reporting rates). Furthermore, the number of reports for sodium citrate (n = 485) was considerably lower than that for NM (n = 1,572). This data imbalance implies that the high signal strengths detected for certain very rare adverse events with sodium citrate (e.g., spinal muscular atrophy with only 4 reports) may lack robustness and be susceptible to false positives. Therefore, future studies are needed to validate these potential signals by pooling data from multiple sources, such as the FDA FAERS database, to confirm these associations. Second, there is a pervasive issue of missing exposure data within VigiAccess. The database does not systematically collect key variables such as the duration of drug exposure, cumulative dose, or time from drug initiation to adverse event onset. Consequently, the adverse event signals identified in this retrospective study are derived solely from the frequency of reported cases and disproportionality metrics (e.g., ROR, PRR, IC, EBGM). These analyses do not allow for an assessment of potential associations between AEs and the length of drug use, nor do they support dose-response or time-to-event interpretations. The absence of detailed dosage, treatment duration, precise indication, and complete concomitant medication history also prevents exploration of dose-response relationships and complicates the accurate assessment of the influence of co-administered drugs. Finally, and most importantly, is the substantial impact of confounding factors. No confounders were included or adjusted for in our statistical tests; all reported signals are unadjusted. In particular, confounding by indication is a major concern. Patients prescribed NM or sodium citrate are typically critically ill with numerous comorbidities. Consequently, many of the detected AEs (e.g., mortality, infections, organ failure) could be strongly linked to the severity of the patients’ underlying conditions rather than being directly caused by the study drugs. In the absence of detailed clinical context and adjustment for these confounders, the detected signals should be interpreted as suggestive statistical associations rather than definitive causal relationships. It is important to emphasize that disproportionality analysis only detects statistical associations, not causal relationships. Extremely high signal values (e.g., ROR >6,000 for citrate toxicity) are often driven by small case numbers (n ≤ 5) and may be substantially influenced by notoriety bias, stimulated reporting, or confounding by indication. Therefore, such findings should be interpreted conservatively as hypothesis-generating signals requiring independent validation, rather than as clinically actionable risk evidence. Our findings are hypothesis-generating only and do not support direct clinical recommendations. Any clinical decision should continue to follow established guidelines and individual patient assessment.

In summary, our study advances the current understanding of NM and sodium citrate safety in several key areas through a systematic analysis of global spontaneous reporting data. Firstly, we have quantified their distinct risk profiles, clarifying NM’s association with immune-mediated shock and sodium citrate’s link to metabolic complications and pregnancy-related exposures. Secondly, we have identified novel potential risk signals, such as “thrombosis in device” for NM and “citrate toxicity” for sodium citrate, providing new foci for pharmacovigilance. Finally, the population-specific insights (regarding age, sex, and region) derived from real-world data offer more targeted perspectives for risk management in different patient subgroups.

## 5 Conclusion

Both NM and sodium citrate showed distinct disproportionality patterns in spontaneous reporting data. These hypothesis-generating signals suggest that immune-related AEs for NM and metabolic complications for sodium citrate warrant further investigation in controlled studies. Particular attention should be paid to potential AEs not listed in the prescribing information. Due to the inherent limitations of spontaneous reporting data, these findings should be interpreted as exploratory and do not constitute evidence-based guidance for clinical practice. Confirmation through independent pharmacoepidemiological databases and prospective studies is required before any clinical recommendations can be made.

## Data Availability

The datasets presented in this study can be found in online repositories. The names of the repository/repositories and accession number(s) can be found below: https://www.vigiaccess.org.
