# Supplementary material for: Original research a real-world study of adverse events of nafamostat mesylate and sodium citrate based on the world health organization-VigiAccess database
Source: Front Pharmacol. 2026 May 20;17:1795980. doi: 10.3389/fphar.2026.1795980 (PMC13230116; doi:10.3389/fphar.2026.1795980)
Supplement: Supplementary file 1 [file Supplementaryfile1.docx]

**Supplemental Materials**

| **Table S1** | **Page 2** |
| --- | --- |
| **Table S2** | **Page 3** |
| **Table S3** | **Page 4-6** |
| **Table S4** | **Page 7-11** |

Table S1. Two-by-two contingency table for disproportionality analysis

| **Item** | **Target AEs** | **Other AEs** | **Total** |
| --- | --- | --- | --- |
| **Target drugs** | a | b | a + b |
| **Other drugs** | c | d | c + d |
| **Total** | a + c | b + d | a + b + c + d |

Table S2. The principles of disproportionate measurement and the criteria for signal detection

| **Method** | **Calculation formula** | **Criteria** |
| --- | --- | --- |
| ROR | $ROR=\frac{a / c}{b / d}$ | a ≥ 3  ROR≥ 1  95%CI (lower limit) > 1 |
|  | $SE(lnROR)=\sqrt{\frac{1}{a}+\frac{1}{b}+\frac{1}{c}+\frac{1}{d}}$ |  |
|  | $95\%CI= e^{\ln\left( ROR \right)\pm1.96se}$ |  |
| PRR | $PRR=\frac{a / (a+b)}{c / (c+d)}$ | a ≥ 3  PRR≥ 2  95%CI (lower limit) > 1 |
|  | $SE(lnPRR)=\sqrt{\frac{1}{a}-\frac{1}{a+b}+\frac{1}{c}-\frac{1}{c+d}}$ |  |
|  | $95\%CI= e^{\ln\left( PRR \right)\pm1.96se}$ |  |
|  | $\chi2 =\frac{{(ad-bc)}^{2}(a+b+c+d)}{( a+b)(a+c)(c+d)(b+d)}$ | a ≥ 3  PRR ≥ 2  $\chi2\geq4$ |
| BCPNN | IC=${log}_{2}\frac{p(x,y)}{p(x)p(y)}={log}_{2}\frac{a(a+b+c+d)}{(a+b)(a+c)}$ | IC025>0 |
|  | E(IC)=${log}_{2}\frac{(a+\gamma11)(a+b+c+d+\alpha)(a+b+c+d+\beta)}{（a+b+c+d+\gamma）(a+b+\alpha1)(a+c+\beta1)}$ |  |
|  | $V\left( IC \right)=\frac{1}{{(ln2)}^{2}}\{\left[ \frac{\left( a+b+c+d \right)-a+\gamma-\gamma11}{\left( a+\gamma11 \right)\left( 1+a+b+c+d+\gamma\right)} \right]+\left[ \frac{\left( a+b+c+d \right)-\left( a+b \right)+\alpha-\alpha1}{\left( a+b+\alpha1 \right)\left( 1+a+b+c+d+\alpha\right)} \right]+\left[ \frac{\left( a+b+c+d \right)-\left( a+c \right)+\beta-\beta1}{\left( a+c+\beta1 \right)\left( 1+a+b+c+d+\beta\right)} \right]\}$ |  |
|  | $\gamma=\gamma11\frac{(a+b+c+d+\alpha)(a+b+c+d+\beta)}{(a+b+\alpha1)(a+c+\beta1)}$ |  |
|  | *IC-2SD=E(IC)-2*$\sqrt{V(IC)}$  $\alpha1=\beta1=1；\alpha=\beta=2；\gamma11=1$ |  |
| EBGM | $EBGM=\frac{a(a+b+c+d)}{\left( a+c \right)(a+b)}$ | EBGM05>2 |
|  | $SE(lnEBGM)=\sqrt{\frac{1}{a}+\frac{1}{b}+\frac{1}{c}+\frac{1}{d}}$ |  |
|  | $95\%CI= e^{\ln\left( EBGM \right)\pm1.96se}$ |  |

Table S3. Signal strength of adverse events at the PT level for nafamostat mesylate ranked by reports

| **System Organ Class(SOC)** | **Preferred Term(PT)** | **Case reports** | **ROR(95% CI)** | **PRR(95% CI)** | **Chi-Square** | **IC(IC025)** | **EBGM (EBGM05)** |
| --- | --- | --- | --- | --- | --- | --- | --- |
| Immune system disorders | Anaphylactic shock | 391 | 143.23(128.51,159.65) | 119.94(109.53,131.33) | 46067.4 | 6.90(6.36) | 119.65(107.34) |
| Skin and subcutaneous tissue disorders | Pruritus | 189 | 5.35(4.61,6.21) | 5.01(4.37,5.74) | 615.77 | 2.32(2.08) | 5.01(4.32) |
| Immune system disorders | Anaphylactic reaction | 121 | 28.57(23.79,34.30) | 27.17(22.84,32.33) | 3054.35 | 4.76(4.21) | 27.16(22.62) |
| Vascular disorders | Shock | 119 | 186.27(154.87,224.05) | 177.04(148.54,210.99) | 20761.4 | 7.46(5.89) | 176.41(146.67) |
| Investigations | Blood pressure decreased | 113 | 50.97(42.20,61.58) | 48.61(40.60,58.20) | 5269.08 | 5.60(4.82) | 48.56(40.20) |
| Respiratory, thoracic and mediastinal disorders | Dyspnoea | 84 | 3.19(2.57,3.97) | 3.11(2.52,3.84) | 121.97 | 1.64(1.28) | 3.11(2.51) |
| Skin and subcutaneous tissue disorders | Urticaria | 57 | 2.67(2.05,3.47) | 2.63(2.04,3.40) | 58.19 | 1.40(0.97) | 2.63(2.02) |
| Vascular disorders | Hypotension | 53 | 6.28(4.78,8.25) | 6.16(4.72,8.04) | 230.03 | 2.62(2.09) | 6.16(4.69) |
| Metabolism and nutrition disorders | Hyperkalaemia | 49 | 39.60(29.84,52.56) | 38.81(29.41,51.21) | 1804.54 | 5.28(4.05) | 38.78(29.22) |
| Metabolism and nutrition disorders | Hyponatraemia | 29 | 13.12(9.10,18.92) | 12.97(9.03,18.63) | 320.62 | 3.70(2.68) | 12.97(8.99) |
| General disorders and administration site conditions | Chest discomfort | 26 | 4.98(3.38,7.33) | 4.94(3.37,7.24) | 81.80 | 2.30(1.55) | 4.94(3.35) |
| Immune system disorders | Anaphylactoid reaction | 19 | 11.63(7.41,18.27) | 11.55(7.38,18.07) | 183.16 | 3.53(2.27) | 11.55(7.35) |
| Product issues | Thrombosis in device | 18 | 264.71(166.28,421.40) | 262.72(165.61,416.78) | 4668.38 | 8.03(3.49) | 261.34(164.16) |
| Nervous system disorders | Depressed level of consciousness | 16 | 12.65(7.74,20.68) | 12.57(7.71,20.49) | 170.46 | 3.65(2.20) | 12.57(7.69) |
| Investigations | Platelet count decreased | 14 | 4.73(2.80,8.00) | 4.71(2.79,7.94) | 40.93 | 2.23(1.17) | 4.71(2.78) |
| Blood and lymphatic system disorders | Eosinophilia | 13 | 14.99(8.69,25.85) | 14.91(8.67,25.64) | 168.73 | 3.90(2.13) | 14.91(8.64) |
| Skin and subcutaneous tissue disorders | Rash pruritic | 13 | 3.60(2.09,6.22) | 3.59(2.09,6.17) | 24.32 | 1.84(0.82) | 3.59(2.08) |
| Investigations | Eosinophil count increased | 11 | 54.68(30.23,98.91) | 54.43(30.18,98.19) | 576.39 | 5.76(2.48) | 54.38(30.06) |
| Skin and subcutaneous tissue disorders | Cold sweat | 9 | 7.20(3.74,13.86) | 7.18(3.74,13.78) | 47.89 | 2.84(1.23) | 7.18(3.73) |
| Gastrointestinal disorders | Retroperitoneal haemorrhage | 9 | 81.95(42.57,157.79) | 81.65(42.51,156.81) | 715.80 | 6.35(2.26) | 81.52(42.34) |
| Cardiac disorders | Cardio-respiratory arrest | 9 | 7.24(3.76,13.93) | 7.21(3.76,13.85) | 48.19 | 2.85(1.24) | 7.21(3.75) |
| Investigations | Oxygen saturation decreased | 8 | 4.97(2.48,9.94) | 4.95(2.48,9.89) | 25.25 | 2.31(0.82) | 4.95(2.47) |
| Infections and infestations | Septic shock | 8 | 9.32(4.66,18.67) | 9.30(4.65,18.57) | 59.24 | 3.22(1.31) | 9.29(4.64) |
| General disorders and administration site conditions | Multiple organ dysfunction syndrome | 8 | 9.52(4.76,19.06) | 9.49(4.75,18.96) | 60.80 | 3.25(1.32) | 9.49(4.74) |
| General disorders and administration site conditions | Injection site rash | 7 | 4.52(2.15,9.49) | 4.51(2.15,9.45) | 19.14 | 2.17(0.63) | 4.51(2.15) |
| Respiratory, thoracic and mediastinal disorders | Respiratory arrest | 6 | 6.08(2.73,13.55) | 6.07(2.73,13.49) | 25.40 | 2.60(0.72) | 6.07(2.72) |
| Nervous system disorders | Altered state of consciousness | 6 | 10.02(4.49,22.32) | 9.99(4.49,22.22) | 48.56 | 3.32(1.04) | 9.99(4.48) |
| Respiratory, thoracic and mediastinal disorders | Acute respiratory distress syndrome | 5 | 11.42(4.75,27.47) | 11.40(4.75,27.36) | 47.43 | 3.51(0.88) | 11.40(4.74) |
| Musculoskeletal and connective tissue disorders | Haematoma muscle | 5 | 53.64(22.29,129.05) | 53.53(22.29,128.55) | 257.46 | 5.74(1.28) | 53.47(22.22) |
| Blood and lymphatic system disorders | Disseminated intravascular coagulation | 5 | 16.26(6.76,39.11) | 16.23(6.76,38.96) | 71.44 | 4.02(1.02) | 16.22(6.75) |
| Vascular disorders | Phlebitis | 4 | 5.65(2.12,15.07) | 5.64(2.12,15.03) | 15.29 | 2.50(0.26) | 5.64(2.12) |
| Injury, poisoning and procedural complications | Post procedural haemorrhage | 4 | 12.71(4.77,33.91) | 12.70(4.77,33.80) | 43.09 | 3.67(0.63) | 12.69(4.76) |
| Vascular disorders | Shock symptom | 4 | 120.41(45.10,321.47) | 120.21(45.10,320.41) | 471.73 | 6.91(0.98) | 119.92(44.92) |
| Investigations | Blood potassium increased | 4 | 9.89(3.71,26.37) | 9.87(3.71,26.28) | 31.89 | 3.30(0.54) | 9.87(3.70) |
| Immune system disorders | Anaphylactoid shock | 4 | 143.45(53.72,383.06) | 143.21(53.71,381.80) | 563.23 | 7.16(0.99) | 142.80(53.47) |
| Investigations | Prothrombin time prolonged | 3 | 10.23(3.30,31.75) | 10.22(3.30,31.67) | 24.95 | 3.35(0.18) | 10.22(3.29) |
| Metabolism and nutrition disorders | Electrolyte imbalance | 3 | 9.31(3.00,28.89) | 9.30(3.00,28.82) | 22.22 | 3.22(0.15) | 9.30(3.00) |
| Respiratory, thoracic and mediastinal disorders | Sputum increased | 3 | 22.47(7.24,69.74) | 22.44(7.24,69.56) | 61.44 | 4.49(0.37) | 22.43(7.23) |
| Investigations | Amylase increased | 3 | 12.96(4.18,40.22) | 12.95(4.18,40.12) | 33.06 | 3.69(0.25) | 12.94(4.17) |
| Investigations | Activated partial thromboplastin time prolonged | 3 | 21.95(7.07,68.13) | 21.93(7.07,67.95) | 59.89 | 4.45(0.37) | 21.92(7.06) |
| Vascular disorders | Shock haemorrhagic | 3 | 14.04(4.52,43.57) | 14.02(4.53,43.46) | 36.28 | 3.81(0.28) | 14.02(4.52) |
| Metabolism and nutrition disorders | Hypercalcaemia | 3 | 8.59(2.77,26.65) | 8.58(2.77,26.58) | 20.08 | 3.10(0.12) | 8.58(2.76) |

Note1:ranked by reports.

Note2:Signals are detected when all the following criteria are met:a ≥ 3, PRR ≥2 and Chi-Square ≥ 4, lower limit of 95% CI of ROR > 1, IC025 > 0, EBGM05 > 2.

Table S4. Signal strength of adverse events at the PT level for Sodium citrate ranked by reports

| **System Organ Class(SOC)** | **Preferred Term(PT)** | **Case reports** | **ROR(95% CI)** | **PRR(95% CI)** | **Chi-Square** | **IC(IC025)** | **EBGM (EBGM05)** |
| --- | --- | --- | --- | --- | --- | --- | --- |
| General disorders and administration site conditions | Death | 46 | 3.58(2.67,4.81) | 3.51(2.64,4.67) | 83.28 | 1.81(1.31) | 3.51(2.62) |
| Injury, poisoning and procedural complications | Exposure during pregnancy | 17 | 10.10(6.26,16.29) | 10.01(6.24,16.06) | 137.93 | 3.32(2.05) | 10.00(6.20) |
| Vascular disorders | Pallor | 16 | 8.42(5.15,13.78) | 8.35(5.13,13.59) | 103.60 | 3.06(1.84) | 8.35(5.10) |
| Cardiac disorders | Cardiac arrest | 14 | 8.28(4.90,14.02) | 8.22(4.88,13.85) | 88.89 | 3.04(1.72) | 8.22(4.86) |
| Injury, poisoning and procedural complications | Foetal exposure during pregnancy | 13 | 10.65(6.17,18.39) | 10.58(6.15,18.18) | 112.79 | 3.40(1.88) | 10.58(6.13) |
| Pregnancy, puerperium and perinatal conditions | Premature baby | 11 | 23.58(13.03,42.66) | 23.42(13.00,42.21) | 236.11 | 4.55(2.19) | 23.42(12.94) |
| Infections and infestations | Sepsis | 9 | 4.73(2.46,9.10) | 4.71(2.45,9.03) | 26.31 | 2.23(0.86) | 4.71(2.44) |
| Injury, poisoning and procedural complications | Maternal exposure during pregnancy | 9 | 6.72(3.49,12.95) | 6.69(3.49,12.84) | 43.61 | 2.74(1.18) | 6.69(3.48) |
| Gastrointestinal disorders | Ascites | 8 | 17.84(8.91,35.75) | 17.76(8.90,35.46) | 126.54 | 4.15(1.67) | 17.76(8.86) |
| Injury, poisoning and procedural complications | Procedural complication | 7 | 72.25(34.38,151.86) | 71.95(34.34,150.73) | 489.27 | 6.17(1.84) | 71.88(34.20) |
| Infections and infestations | Pneumonia aspiration | 7 | 21.48(10.22,45.13) | 21.39(10.21,44.80) | 136.04 | 4.42(1.57) | 21.38(10.18) |
| Respiratory, thoracic and mediastinal disorders | Respiratory arrest | 7 | 10.44(4.97,21.93) | 10.40(4.96,21.78) | 59.47 | 3.38(1.23) | 10.40(4.95) |
| General disorders and administration site conditions | Multiple organ dysfunction syndrome | 7 | 12.25(5.83,25.74) | 12.20(5.83,25.56) | 72.00 | 3.61(1.32) | 12.20(5.81) |
| Injury, poisoning and procedural complications | Post procedural complication | 7 | 29.36(13.97,61.69) | 29.24(13.96,61.24) | 190.85 | 4.87(1.67) | 29.23(13.91) |
| Product issues | Device infusion issue | 7 | 212.41(100.99,446.75) | 211.50(100.88,443.42) | 1462.30 | 7.72(1.93) | 210.89(100.27) |
| General disorders and administration site conditions | Inflammation | 6 | 5.42(2.43,12.08) | 5.40(2.43,12.01) | 21.53 | 2.43(0.64) | 5.40(2.42) |
| Pregnancy, puerperium and perinatal conditions | Live birth | 6 | 51.66(23.17,115.19) | 51.47(23.15,114.43) | 296.76 | 5.68(1.55) | 51.44(23.07) |
| Nervous system disorders | Unresponsive to stimuli | 6 | 8.61(3.86,19.20) | 8.58(3.86,19.08) | 40.21 | 3.10(0.95) | 8.58(3.85) |
| Vascular disorders | Circulatory collapse | 6 | 10.21(4.58,22.76) | 10.18(4.58,22.62) | 49.66 | 3.35(1.05) | 10.18(4.56) |
| Psychiatric disorders | Stress | 6 | 5.67(2.55,12.65) | 5.66(2.54,12.57) | 23.01 | 2.50(0.67) | 5.66(2.54) |
| Nervous system disorders | Generalised tonic-clonic seizure | 5 | 7.18(2.98,17.27) | 7.16(2.98,17.17) | 26.50 | 2.84(0.64) | 7.16(2.97) |
| Metabolism and nutrition disorders | Hypocalcaemia | 5 | 15.69(6.52,37.76) | 15.65(6.52,37.55) | 68.56 | 3.97(1.00) | 15.64(6.50) |
| Injury, poisoning and procedural complications | Citrate toxicity | 5 | 6740.61(2692.95,16872.1) | 6719.87(2691.61,16776.8) | 30742.8 | 12.59(1.35) | 6150.47(2457.18) |
| Infections and infestations | Appendicitis | 5 | 31.15(12.95,74.96) | 31.06(12.94,74.54) | 145.41 | 4.96(1.19) | 31.05(12.90) |
| Cardiac disorders | Cardiogenic shock | 5 | 26.25(10.91,63.16) | 26.17(10.91,62.80) | 121.02 | 4.71(1.15) | 26.16(10.87) |
| Respiratory, thoracic and mediastinal disorders | Tachypnoea | 5 | 7.89(3.28,18.98) | 7.87(3.28,18.87) | 29.97 | 2.98(0.69) | 7.86(3.27) |
| Respiratory, thoracic and mediastinal disorders | Atelectasis | 5 | 27.59(11.47,66.39) | 27.51(11.46,66.02) | 127.70 | 4.78(1.16) | 27.50(11.43) |
| Gastrointestinal disorders | Appendicolith | 5 | 898.74(371.56,2173.89) | 895.98(371.42,2161.40) | 4415.42 | 9.79(1.39) | 885.07(365.91) |
| Infections and infestations | Respiratory tract infection bacterial | 4 | 506.99(189.40,1357.10) | 505.75(189.39,1350.51) | 2001.06 | 8.97(1.01) | 502.25(187.63) |
| Nervous system disorders | Brain injury | 4 | 23.03(8.63,61.44) | 22.97(8.63,61.14) | 84.04 | 4.52(0.80) | 22.97(8.61) |
| Nervous system disorders | Motor dysfunction | 4 | 21.81(8.17,58.18) | 21.75(8.17,57.90) | 79.19 | 4.44(0.79) | 21.75(8.15) |
| Nervous system disorders | Ischaemic cerebral infarction | 4 | 163.86(61.36,437.60) | 163.46(61.35,435.48) | 644.41 | 7.35(0.99) | 163.09(61.07) |
| Metabolism and nutrition disorders | Metabolic disorder | 4 | 53.00(19.86,141.43) | 52.87(19.86,140.74) | 203.41 | 5.72(0.92) | 52.83(19.80) |
| Injury, poisoning and procedural complications | Maternal exposure during delivery | 4 | 131.09(49.10,350.00) | 130.77(49.09,348.31) | 514.17 | 7.03(0.98) | 130.53(48.89) |
| Respiratory, thoracic and mediastinal disorders | Increased bronchial secretion | 4 | 103.30(38.70,275.77) | 103.05(38.70,274.44) | 403.68 | 6.69(0.97) | 102.91(38.55) |
| General disorders and administration site conditions | Disease complication | 4 | 73.45(27.52,196.04) | 73.27(27.52,195.09) | 284.86 | 6.19(0.95) | 73.20(27.43) |
| Respiratory, thoracic and mediastinal disorders | Lung consolidation | 4 | 86.40(32.37,230.63) | 86.19(32.37,229.51) | 336.43 | 6.43(0.96) | 86.09(32.25) |
| Congenital, familial and genetic disorders | Spinal muscular atrophy | 4 | 665.94(248.52,1784.46) | 664.30(248.50,1775.80) | 2625.18 | 9.36(1.01) | 658.28(245.66) |
| Investigations | Chest X-ray abnormal | 4 | 23.56(8.83,62.86) | 23.51(8.83,62.56) | 86.18 | 4.55(0.80) | 23.50(8.81) |
| Respiratory, thoracic and mediastinal disorders | Sputum increased | 4 | 44.07(16.52,117.60) | 43.96(16.52,117.04) | 167.86 | 5.46(0.90) | 43.94(16.47) |
| Investigations | Blood urine present | 4 | 12.22(4.58,32.60) | 12.19(4.58,32.44) | 41.09 | 3.61(0.62) | 12.19(4.57) |
| Gastrointestinal disorders | Paraesthesia oral | 4 | 6.23(2.33,16.62) | 6.22(2.34,16.54) | 17.51 | 2.64(0.31) | 6.22(2.33) |
| Pregnancy, puerperium and perinatal conditions | Premature labour | 4 | 42.23(15.83,112.68) | 42.13(15.83,112.14) | 160.52 | 5.40(0.90) | 42.10(15.78) |
| Nervous system disorders | Cerebral ischaemia | 4 | 40.32(15.11,107.59) | 40.22(15.11,107.08) | 152.92 | 5.33(0.89) | 40.20(15.07) |
| Gastrointestinal disorders | Intestinal ischaemia | 4 | 50.29(18.85,134.21) | 50.17(18.85,133.57) | 192.65 | 5.65(0.92) | 50.14(18.79) |
| Pregnancy, puerperium and perinatal conditions | Premature delivery | 4 | 17.83(6.68,47.58) | 17.79(6.69,47.36) | 63.39 | 4.15(0.74) | 17.79(6.67) |
| Infections and infestations | Pneumococcal sepsis | 4 | 249.15(93.24,665.78) | 248.54(93.24,662.54) | 982.83 | 7.95(1.00) | 247.70(92.70) |
| Surgical and medical procedures | Resuscitation | 4 | 55.29(20.72,147.56) | 55.16(20.72,146.84) | 212.55 | 5.78(0.93) | 55.12(20.65) |
| Surgical and medical procedures | Caesarean section | 4 | 30.52(11.44,81.42) | 30.44(11.44,81.03) | 113.86 | 4.93(0.85) | 30.43(11.40) |
| Investigations | Heart rate decreased | 4 | 7.59(2.85,20.25) | 7.57(2.85,20.16) | 22.83 | 2.92(0.42) | 7.57(2.84) |
| Pregnancy, puerperium and perinatal conditions | Normal newborn | 4 | 41.79(15.66,111.52) | 41.69(15.66,110.98) | 158.78 | 5.38(0.90) | 41.67(15.62) |
| Cardiac disorders | Ventricular fibrillation | 4 | 17.22(6.45,45.93) | 17.18(6.45,45.71) | 60.93 | 4.10(0.73) | 17.17(6.44) |
| Nervous system disorders | Motor neurone disease | 3 | 320.77(103.09,998.12) | 320.18(103.11,994.21) | 950.37 | 8.32(0.54) | 318.78(102.45) |
| Investigations | Blood phosphorus increased | 3 | 75.82(24.41,235.46) | 75.68(24.42,234.54) | 220.85 | 6.24(0.50) | 75.60(24.34) |
| Respiratory, thoracic and mediastinal disorders | Lung infiltration | 3 | 13.24(4.26,41.09) | 13.22(4.27,40.94) | 33.87 | 3.72(0.26) | 13.21(4.26) |
| Nervous system disorders | Demyelination | 3 | 35.77(11.52,111.05) | 35.70(11.52,110.62) | 101.15 | 5.16(0.44) | 35.69(11.49) |
| Surgical and medical procedures | Lymphadenectomy | 3 | 172.43(55.48,535.92) | 172.11(55.49,533.82) | 509.16 | 7.42(0.53) | 171.71(55.25) |
| Metabolism and nutrition disorders | Metabolic acidosis | 3 | 7.98(2.57,24.77) | 7.97(2.57,24.68) | 18.28 | 2.99(0.09) | 7.97(2.57) |
| Musculoskeletal and connective tissue disorders | Muscle atrophy | 3 | 14.35(4.62,44.53) | 14.32(4.62,44.36) | 37.17 | 3.84(0.28) | 14.32(4.61) |
| Gastrointestinal disorders | Frequent bowel movements | 3 | 7.99(2.58,24.81) | 7.98(2.58,24.72) | 18.32 | 3.00(0.09) | 7.98(2.57) |
| Renal and urinary disorders | Micturition urgency | 3 | 12.20(3.93,37.86) | 12.18(3.93,37.72) | 30.77 | 3.61(0.24) | 12.17(3.92) |
| Investigations | Blood calcium decreased | 3 | 16.69(5.38,51.80) | 16.66(5.38,51.60) | 44.15 | 4.06(0.32) | 16.65(5.37) |
| Musculoskeletal and connective tissue disorders | Scoliosis | 3 | 30.74(9.90,95.44) | 30.69(9.90,95.07) | 86.13 | 4.94(0.42) | 30.67(9.88) |
| Respiratory, thoracic and mediastinal disorders | Hyperventilation | 3 | 13.60(4.38,42.23) | 13.58(4.38,42.07) | 34.96 | 3.76(0.27) | 13.58(4.37) |
| Vascular disorders | Air embolism | 3 | 460.18(147.73,1433.46) | 459.33(147.77,1427.84) | 1363.38 | 8.83(0.54) | 456.45(146.53) |
| Surgical and medical procedures | Stoma closure | 3 | 1246.44(397.72,3906.30) | 1244.14(397.81,3891.01) | 3663.61 | 10.26(0.54) | 1223.18(390.30) |
| Product issues | Product taste abnormal | 3 | 13.05(4.20,40.50) | 13.02(4.20,40.34) | 33.30 | 3.70(0.26) | 13.02(4.19) |
| Respiratory, thoracic and mediastinal disorders | Bronchial disorder | 3 | 131.48(42.32,408.51) | 131.24(42.33,406.91) | 387.04 | 7.03(0.52) | 131.00(42.16) |

Note1:ranked by reports.

Note2:Signals are detected when all the following criteria are met:a ≥ 3, PRR ≥2 and Chi-Square ≥ 4, lower limit of 95% CI of ROR > 1, IC025 > 0, EBGM05 > 2.
